# Proposed physical mechanism that gives rise to cosmic inflation

**DOI:** 10.1038/s41598-023-49106-0

**Published:** 2023-12-09

**Authors:** Bruce M. Law

**Affiliations:** Department of Physics, Kansas State University, 116 Cardwell Hall, Manhattan, KS 66506-2601 USA

**Keywords:** Cosmology, Early universe, Phase transitions and critical phenomena

## Abstract

Early in the Universe a chemical equilibrium exists between photons and electron–positron ($e^{ - } e^{ + }$) pairs. In the electron Born self-energy (eBse) model the $e^{ - } e^{ + }$ plasma falls out of equilibrium above a glass transition temperature $T_{G} = 1.06 \times 10^{17} K$ determined by the maximum electron/positron number density of $1/(2R_{e} )^{3}$ where $R_{e}$ is the electron radius. In the glassy phase ($T > T_{G}$) the Universe undergoes exponential acceleration, characteristic of cosmic inflation, with a constant potential energy density $\psi_{G} = 1.9 \times 10^{50} J/m^{3}$. At lower temperatures $T < T_{G}$ photon-$e^{ - } e^{ + }$ chemical equilibrium is restored and the glassy phase gracefully exits to the $\Lambda CDM$ cosmological model when the equation of state $w = 1/3$, corresponding to a cross-over temperature $T_{X} = 0.94 \times 10^{17} K$. In the eBse model the inflaton scalar field is temperature $T$ where the potential energy density $\psi (T)$ is a plateau potential, in agreement with Planck collaboration 2013 findings. There are no free parameters that require fine tuning to give cosmic inflation in the eBse model.

## Introduction

**Figure 1:**
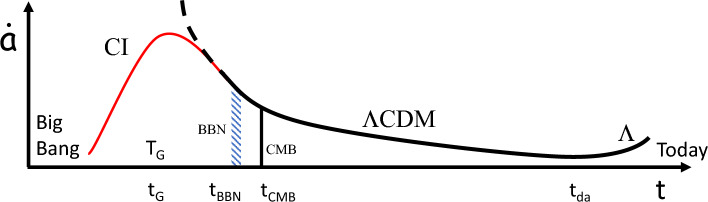
Variation in scale factor velocity $\dot{a}$ versus cosmic time $t$. Big Bang Nucleosynthesis (BBN) at time $t_{BBN} \sim 1{\text{s}} - 5\min$, Cosmic Microwave Background (CMB) at $t_{CMB} \sim 380,000{\text{yr}}$, the deceleration-acceleration transition at $t_{da} \sim 7{\text{Gyr}}$, the cosmological constant $\Lambda$ causes accelerated expansion. $\Lambda CDM$ model: black dashed and solid lines. Cosmic inflation (CI): red solid line. eBse model: exponential acceleration, during CI, terminates at a glass transition temperature $T_{G}$ at time $t_{G} \sim 3 \times 10^{ - 11} {\text{s}}$.

The current cosmological paradigm for the expansion of the Universe contains many unexplained mysteries and consists of two adjoining theories: cosmic inflation (CI, Fig. 1 red curve)^1–3^, at early times $t$, that joins smoothly onto the $\Lambda CDM$ model (Fig. 1 black dashed and solid curves)^3–5^ at late times where $\dot{a}$, in Fig. 1, is the expansion or scale factor velocity. The $\Lambda CDM$ model accounts for Big Bang Nucleosynthesis (BBN, the creation of light elements in the early Universe), the existence of a cosmic microwave background (CMB), and cold dark matter (CDM), as well as, a period of accelerated expansion due to Dark Energy (DE) or, equivalently, the cosmological constant $\Lambda$ at late times^4^. For most of the $\Lambda CDM$ model $\dot{a}$ is decreasing with increasing $t$ corresponding to a decelerating expansion due to the attractive nature of gravity. Only recently for $t > t_{da}$, where $t_{da}$ is the transition time from deceleration to acceleration^6^, does $\dot{a}$ increase with increasing $t$, corresponding to an accelerating expansion due to $\Lambda$^3–5^. The $\Lambda CDM$ model results in the following composition for the Universe: ~ 5% ordinary matter (baryons), ~ 25% CDM, and ~ 70% DE.

The CMB is remarkably homogeneous and isotropic with thermal fluctuations $\delta T/T\sim 10^{ - 5} - 10^{ - 4}$ in causally disconnected regions (the Horizon problem) where, additionally, the initial scale factor velocities $\dot{a}_{i}$ are also very homogeneous in causally disconnected regions (the Flatness problem)^3,7^. A method that solves both the Horizon and Flatness problems is for there to be a period of exponential acceleration, or cosmic inflation, that precedes the $\Lambda CDM$ phase and which joins smoothly onto the $\Lambda CDM$ phase (a Graceful Exit). CI is now the accepted paradigm in cosmology because it so elegantly solves both the Horizon and Flatness problems. There are many unknowns in this description of the Universe. What is CDM? What is DE? What gives rise to CI?

In earlier work^8,9^ the author proposed a model, based upon the electron Born self-energy (eBse), that quantitatively explains many astrophysical observations attributed to DE with no adjustable parameters. In this model the electron is assumed to possess a finite, non-zero radius given by^8,10^

1 $$R_{e} = \hbar c/\varepsilon = 1.9 \times 10^{ - 20} m$$

where $\varepsilon = 10.3\;TeV$^11^ is the contact interaction energy between electron–positron collisions at the LEP (large electron–positron collider). Equation (1) arises from the assumption that the relativistic energy $\varepsilon = c\sqrt {p^{2} + m_{e}^{2} c^{2} } \approx cp$, at these high collision energies, where the momentum $p = \hbar /\lambda \approx \hbar /R_{e}$. Here $m_{e}$, $c$, $\hbar$, and $\lambda$ are, respectively, the electron rest mass, speed of light in a vacuum, reduced Planck’s constant, and electron wavelength at energy $\varepsilon$.

In Quantum Electrodynamics (QED) the electron is assumed to be a point particle ($R_{e} \to 0$), thus, Eq. (1) would represent an upper bound to the electron radius within QED (namely, the actual electron radius would be less than this experimental estimate). Unfortunately, this point particle assumption for the electron (and the resultant mass renormalization to eliminate divergences) leads to a number of fundamental difficulties in QED which are not well recognized and are rarely discussed. Specifically, the non-local energy is not conserved for the electron where, in addition, the treatment of electrons and other charged particles are inconsistent with each other. These inconsistencies within Physics can only be resolved if the electron possesses a finite, non-zero radius, as is assumed in the eBse model. For any assumed electron radius, such as in Eq. (1), a necessary requirement is that this radius not produce any conflicts between theory and experiment within QED. These issues, and the interrelationship between QED and the eBse model, are discussed in the Supplementary Material.

The eBse description of DE is applicable to cosmological phenomena occurring at late times and small redshifts ($z\sim 0 - 2$). Will a finite-sized electron have any other cosmological consequences and are these consequences consistent with astrophysical measurements? In particular, the finite-size of an electron is likely to have a significant impact at very high densities (in the CI phase) when the separation distance between neighboring electrons and positrons is of order $2R_{e}$. The purpose of this current publication is to explore this ultrahigh density regime. We find that the eBse model in this region exhibits exponential acceleration, due to a constant potential energy density, in agreement with the expectations for CI.

This publication is set out as follows. The $\Lambda CDM$ and cosmic inflation models are outlined in Section “$\Lambda CDM$ and cosmic inflation models”. Section “Electron Born self-energy model at ultrahigh densities” discusses the eBse model at ultrahigh densities. This publication concludes with a discussion in Section “Discussion”. The eBse model is an extension of QED. The interrelationship between the eBse model and QED is described in the Supplementary Material.

## $\Lambda CDM$ and cosmic inflation models

The cosmological expansion of the Universe is described by Einstein’s General Theory of Relativity (GR) which relates the space–time metric $g_{\mu \nu }$ to the energy–momentum tensor $T_{\mu \nu }$. If the Universe is homogeneous and isotropic, as is normally assumed during cosmic expansion, then the GR equations reduce to the Friedmann equations given below. A pedagogical description of this interrelationship can be found in^12^ (Chapter 3). The expansion, during the $\Lambda CDM$ phase, is usually described by the Friedmann equation for the scale factor velocity

2 $$H^{2} = \left( {\frac{{\dot{a}}}{a}} \right)^{2} = \frac{8\pi G}{{3c^{2} }}\Pi^{tot} - \frac{{\kappa c^{2} }}{{a^{2} }},$$

where $H$ is Hubble’s parameter, $a$ the scale factor, $\kappa$ the spatial curvature, and $G$ Newton’s gravitational constant. The expansion in Eq. (2) is driven by the total energy density of intergalactic space $\Pi^{tot}$ where, in the $\Lambda CDM$ model,

3 $$\Pi^{tot} = \frac{{\Pi^{R} }}{{a^{4} }} + \frac{{\Pi^{B} + \Pi^{CDM} }}{{a^{3} }} + \frac{{\Pi^{DE} }}{{a^{3(1 + w)} }},$$

has contributions from radiation ($R$), baryons ($B$), $CDM$, and $DE$, and the equation of state $w = P/\Pi ( \approx - 1)$ is the ratio of pressure $P$ to energy density $\Pi$.

Rather than considering the scale factor velocity, as in Eq. (2), a useful alternative is to consider the Friedmann equation for the scale factor acceleration $\ddot{a}$ which takes the form

4 $$\frac{{\ddot{a}}}{a} = - \frac{4\pi G}{{3c^{2} }}(\Pi + 3P).$$

According to Eq. (4) if $w = P/\Pi > - 1/3$ ($< - 1/3$) then the Universe decelerates (accelerates) because $\ddot{a} < 0$ ($\ddot{a} > 0$). In the $\Lambda CDM$ model, at late times ($t > t_{da}$), where the expansion of the Universe is accelerating due to DE, astrophysical measurements indicate that $w \approx - 1$^13,14^. In CI the acceleration of the Universe is also believed to be caused by $w = - 1$ where, to obtain this value for $w$, the inflaton $\varphi$, a scalar field of unknown origin, is modeled as a classical scalar field. For a generic homogeneous scalar field $\varphi$ one can readily show^12^ (p. 164) that the energy–momentum tensor takes the form $T^{\alpha }_{\beta } = - \delta^{\alpha }_{0} \delta^{0}_{\beta } \dot{\varphi }^{2} + \delta^{\alpha }_{\beta } \left[ {\frac{1}{2}\dot{\varphi }^{2} - \psi (\varphi )} \right]$ where $K = \dot{\varphi }^{2} /2$ and $\psi = \psi (\varphi )$ are, respectively, the kinetic energy density and potential energy density of the scalar field, $\delta^{\alpha }_{\beta }$ is the Kronecker delta, and $\alpha ,\;\beta = 0,1,2,3$. Hence, as the energy density $\Pi = - T^{0}_{0}$ where $T^{0}_{0}$ is the time-time component, therefore,

5 $$\Pi = K + \psi.$$

Similarly, as the pressure $P = T^{i}_{i}$ where $T^{i}_{i}$ is the diagonal space-space component (which is the same for $i = 1,2,3$), therefore,

6 $$P = K - \psi.$$

During the inflationary phase

7 $$K < < \psi$$

and, consequently, $$w \equiv P/\Pi = - 1$$.

In traditional inflationary theory a form for $\psi (\varphi )$ is surmised and then various parameters within this potential are fined tuned such that Eq. (7) holds for a time period of^15^

8 $$\sim {5}0 - {6}0\;\tau_{CI} ,$$

where $\tau_{CI}$ is the characteristic time for CI. These requirements on the time scale of the exponential acceleration ensure that the CMB is sufficiently homogeneous and isotropic where, additionally, the flatness of the Universe is also guaranteed. Following the accelerated expansion, the issue for each $\psi (\varphi )$ is, how does the accelerated expansion phase end where the inflaton energy is converted into energy associated with standard particle physics, in thermal equilibrium, so that the $\Lambda CDM$ model can proceed? As different regions of the Universe are expected to exit CI at different time periods this gives rise to the Multiverse—causally disconnected Universes, each of which may possess differing physical constants. Equations (5)–(7) indicate that CI represents a strongly interacting solid phase, early in the Universe, whereas, Eq. (3) indicates that the $\Lambda CDM$ model is a description of non-interacting free particle motion later in the Universe (as a potential energy term between particles is absent).

Astrophysical measurements of thermal fluctuations in the CMB allow one to evaluate how well a particular $\psi (\varphi )$ describes the CMB anisotropy. These CMB measurements indicate that the inflationary phase is best described by a single scalar field possessing a plateau potential^16,17^, namely, $\psi (\varphi )$ is a very flat function of $\varphi$ that ends precipitously at the end of the inflationary period.

## Electron Born self-energy model at ultrahigh densities

### Description of the physics, but without any equations

As much of the Physics, in Section “Electron Born self-energy model at ultrahigh densities”, may be foreign to astrophysicists and cosmologists, the Physics is described in words first, in the absence of any equations, as a guide to the reader. All of the arguments that arise come from soft matter physics, or condensed matter physics, in the vicinity of a glass transition. Due to the ultrahigh densities that are involved, where the average separation distance between particles is of order $2R_{e}$, soft matter physics concepts are necessary in order to describe the physics correctly.

For energies above $1MeV$ there is a chemical equilibrium between the number of photons and the number of electron–positron ($e^{ - } e^{ + }$) pairs in a given volume $V$. This chemical equilibrium varies with temperature $T$. At higher and higher temperatures there are more photons, as well as, more $e^{ - } e^{ + }$ pairs in volume $V$, namely, their number densities increase with increasing temperature. In the eBse model, as both the electron and positron possess a finite, non-zero radius, there will be a maximum number density, specifically, it is impossible to pack more than one electron (or positron) in a volume $V = (2R_{e} )^{3}$ and therefore the maximum electron/positron number density is $1/(2R_{e} )^{3}$. This maximum number density occurs at a glass transition temperature $T_{G}$ (see Eq. (14)) where the electrons and positrons are packed as closely together as is physically possible, given their size (i.e. they are physically in contact with each other). Of course, the packing will be random because $e^{ - } e^{ + }$ pair creation is random, hence, this solid phase will be random, namely, it will be a glass. For temperatures above $T_{G}$ the number density of photons can increase (because a photon is a boson), however, the number density of electrons and positrons will remain fixed at this maximum value. Hence, for $T > T_{G}$, the photon-$e^{ - } e^{ + }$ process falls out of chemical equilibrium where there are too few $e^{ - } e^{ + }$ pairs compared with the number of photons. What this means is that provided the $e^{ - } e^{ + }$ pair creation process is sufficiently fast, compared with the expansion rate of the Universe, then the number density of electrons and positrons remains fixed at $1/(2R_{e} )^{3}$ for $T > T_{G}$. A fixed $e^{ - } /e^{ + }$ number density implies a constant potential energy density $\psi$ for $T > T_{G}$. A constant $\psi$, in cosmology, leads to an exponential acceleration of the Universe as described in Section “Electron–positron glass transition ”. The specific value for this constant $\psi$ (denoted $\psi_{G}$ in Section “Potential energy density in the glassy phase”) can be estimated using standard arguments from solid state physics by noting that the number density in a *random* glassy phase is similar to the number density in a *crystalline* cubic phase. Hence, as the number densities are similar, therefore, the potential energy densities are also likely to be similar. The potential energy density $\psi_{G}$ is therefore estimated by calculating this quantity for an ordered cubic phase of positive and negative charges (the positrons and electrons); this calculation is identical to the calculation of the potential energy density in sodium chloride table salt (see Eqs. (18)–(19)).

**Figure 2:**
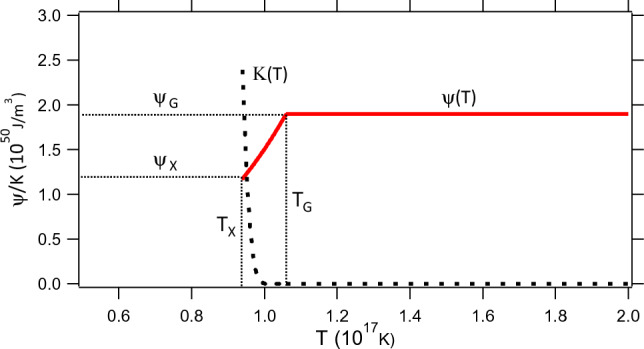
Potential energy density $\psi$ (red solid line) and kinetic energy density $K$ (black dashed line) versus temperature $T$. Cross-over to the $\Lambda CDM$ model occurs at $T_{X}$, a glass transition occurs at $T_{G}$, and cosmic inflation occurs for $T \ge T_{G}$ with $\psi_{G} = \psi (T_{G} )$. The potential energy density $\psi$ is a plateau potential where $\psi$ is constant for $T \ge T_{G}$.

For $T < T_{G}$ the average particle separation distance, $l$, will be greater than $2R_{e}$ and at these lower temperatures photon-$e^{ - } e^{ + }$ chemical equilibrium is restored. The temperature dependence of the potential energy density $\psi (T)$, below the glass transition, can be calculated by an appropriate distance rescaling (see Eqs. (25)–(26)). The kinetic energy density $K$, that appears in Eqs. (5) and (6), can also be estimated by using arguments from colloidal particle physics in the vicinity of a glass transition. As the density of particles is very high an individual particle will experience Brownian motion which is characterized by a diffusion coefficient $D$ and a viscosity $\eta$ (see Eq. (27)) where the viscosity is divergent upon approaching a glass transition (Eq. (29)). Straightforward arguments allow one to calculate the temperature dependence of the kinetic energy density $K(T)$ (see Eqs. (28)–(32)). Once both $\psi (T)$ and $K(T)$, for $T < T_{G}$, are known then various transition points can be determined. The most important transition point is the transition to the $\Lambda CDM$ model which will occur for an equation of state $w = 1/3$, which corresponds to the equation of state for both photons and relativistic fermions. The $w = 1/3$ requirement enables one to determine both the temperature, as well as, the potential energy density where the glassy phase transitions to the $\Lambda CDM$ model (Eqs. (39)–(40)). A plot of $\psi (T)$ and $K(T)$, for the eBse model, is provided in Fig. 2. Sections “Electron–positron glass transition” – “[Sec Sec6]” convert this description, in words, into a mathematical description.

### Electron–positron glass transition

In the early universe, before Recombination, the Universe consists of an ionized plasma of photons, electrons, positrons, protons, anti-protons, and all the other particles of the Standard Model. The Universe also consists of a significant proportion of CDM, however, DE is thought to have played a negligible role. In the following the behavior of electrons and positrons is traced back to earlier and earlier times. At a temperature of $T = 1.2 \times 10^{10} K$, corresponding to an energy of $1MeV$, the conversion of photons to electron–positron pairs first makes its appearance

9 $$\gamma \leftrightarrow e^{ - } + e^{ + }.$$

The $\gamma$ in this equation represents one or more photons. In the Breit-Wheeler process^18^ two photons are converted to an $e^{ - } e^{ + }$ pair in order to conserve both energy and momentum. However, in the presence of a strong electric field (eg. that of a neighboring electron) one photon can be converted to an electron and positron. The later process is called triplet production^19^. The Breit-Wheeler process is exceptionally rare and $e^{ - } e^{ + }$ pair creation normally occurs via triplet production.

The equilibrium process in Eq. (9) can be viewed as a chemical reaction where, because the pair production process is so prolific, the number of electrons $N^{ - }$ is to a good approximation equal to the number of positrons $N^{ + }$. The chemical potential of a photon $\mu = 0$ and, for the current situation, the chemical potential for both the electron and positron is also zero $\mu^{ - } = \mu^{ + } = 0$^20^. The number of electrons or positrons in volume $V$ is given by an integral over the momentum $p$^20^

10 $$N^{ - } = N^{ + } = \frac{V}{{\pi^{2} \hbar^{3} }}\int\limits_{0}^{\infty } {\frac{{p^{2} dp}}{{e^{{\varepsilon /k_{B} T}} + 1}}}.$$

We are most interested in the situation at very high temperatures $T > > m_{e} c^{2} /k_{B}$, where the relativistic energy $\varepsilon = c\sqrt {p^{2} + m_{e}^{2} c^{2} } \approx cp$, and therefore Eq. (10) reduces to^20^ (p. 316)

11 $$N^{ - } = N^{ + } = 0.183\;(k_{B} T/\hbar c)^{3} V.$$

The number of photons in volume $V$ is given by^20^ (p. 187)

12 $$N^{\gamma } = 0.244\;(k_{B} T/\hbar c)^{3} V.$$

At earlier and earlier times, corresponding to higher and higher temperatures, the number of photons, electrons, and positrons increases within volume $V$. In QED where the electron and positron are assumed to be point particles, photon and fermion gases can be taken to arbitrarily high temperatures with no restriction on their densities. However, if electrons and positrons possess a finite, non-zero radius $R_{e}$ (Eq. (1)), then there will be a ***maximum*** number density given by

13 $$(N^{ \pm } /V)_{\max } = 1/(2R_{e} )^{3}.$$

According to Eqs. (11) and (13) this maximum number density is reached at a glass transition temperature of

14 $$T_{G} = \frac{\hbar c}{{2(0.183)^{1/3} R_{e} k_{B} }} = 1.06 \times 10^{17} K,$$

corresponding to an energy of $E_{G} = 9.1\;\,TeV$. For temperatures $T > T_{G}$ this photon-$e^{ - } e^{ + }$ system falls out of equilibrium; namely, in volume $V$, although the number of photons can increase to an arbitrarily large number in accordance with Eq. (12) (because the photon is a boson), the number density of electrons/positrons is restricted to the value given in Eq. (13) (as these particles are fermions). Thus, as the temperature increases the number density of photons increases, whereas, the number density of electrons and positrons remains constant. In this non-equilibrium situation, when the Universe expands and cools the average number density of electrons and positrons decreases. However, locally, at the level of electrons and positrons the number density is controlled by Eqs. (11) and (12) and the system realizes that it’s not in chemical equilibrium (there are too few electrons and positrons) and the number density of electrons and positrons increases to its maximum value given by Eq. (13). In this constant density electron/positron glassy phase the potential energy dominates the kinetic energy because the electrons and positrons are restricted by the Pauli exclusion principle from moving into any neighboring spaces. The physics of this glassy phase will be very different compared with lower temperatures $T < < T_{G}$ where electrons and positrons are free to move as an ideal degenerate relativistic fermi gas.

In this glassy phase where $K \approx 0$ and $\psi$ is constant, for a flat Universe ($\kappa = 0$), Eqs. (2) and (5) have solution

15 $$a(t) = a_{P} \exp \left[ {\frac{{t - t_{P} }}{{\tau_{CI} }}} \right]$$

where $a_{P}$ is the scale factor at Planck time $t_{P}$, while the CI time scale

16 $$\tau_{CI} = \left( {8\pi G\psi /3c^{2} } \right)^{ - 1/2}.$$

Equation (15) allows one to estimate the number of e-folds at the glass transition time $t_{G}$, corresponding to the glass transition temperature $T_{G}$,

17 $$\frac{{t_{G} - t_{P} }}{{\tau_{CI} }} = \ln \left( {\frac{{a_{G} }}{{a_{P} }}} \right) = \ln \left( {\frac{{T_{P} }}{{T_{G} }}} \right) = 34.83.$$

In this calculation it has been assumed that $a\sim 1/T$ (which arises from $VT^{3} = const$ for an adiabatic expansion) is valid up to the Planck temperature^3^. In the glassy phase this relationship may no longer hold because photons experience significant scattering and therefore obey the diffusion equation rather than the wave equation^21^. Future considerations may need to improve upon this assumption.

### *Potential energy density*$\psi$*in the glassy phase*

In the glassy phase $\psi (\varphi )$ can be estimated by assuming that the potential energy density for a *random* close-packed phase of electrons and positrons possesses a similar potential energy density as an *ordered* close-packed crystalline cubic phase of alternating positive and negative charges. This approximation is expected to be reasonable because the packing fraction for cubic packing $0.52$^22^ (p. 16) is similar to the packing fraction for a random loose packed glassy phase $0.56$^23^. For a crystalline structure the electrical potential at site $r_{i}$ is given by^22^

18 $$V_{i} = \frac{q}{{4\pi \varepsilon_{o} }}\sum\limits_{j \ne i} {\frac{{z_{j} }}{{r_{ij} }}} = \frac{q}{{4\pi \varepsilon_{o} r_{o} }}M$$

where the summation is over sites $j$ at coordinate $\vec{r}_{j}$, $z_{j}$ is the sign (+ or -) of the $jth$ charge, and the separation distance $r_{ij} = \left| {\vec{r}_{i} - \vec{r}_{j} } \right|$, the nearest neighbor distance $r_{o} = 2R_{e}$, $q$ is the charge, and $M$ is the Madelung constant that depends upon the crystallographic structure. For a cubic crystal $M = 1.75$^22^ (p. 91). As the energy of an electron at site $i$ is $U_{i} = qV_{i}$, therefore, the total potential energy density is

19 $$\psi_{G} = \frac{{q^{2} M}}{{8\pi \varepsilon_{o} R_{e} (2R_{e} )^{3} }} = 1.9 \times 10^{50} J/m^{3}.$$

Equation (19) assumes that the “hard sphere” interaction dominates and that there is insufficient room for both a spin up and spin down electron at site $i$. If spin up and spin down electrons can be including at site $i$ then one should multiply Eq. (19) by a factor of 4 (because there would be a charge of $2q$ at each site). Equations (18)–(19) are identical to the calculation of the potential energy density for ordinary table salt, sodium chloride, which possesses a crystalline cubic structure.

A Coulomb potential has been assumed in the sum in Eq. (18) without any accounting for virtual electrons and positrons that may screen the charge. At nearest neighbor separation distances of $2R_{e}$, between an electron and positron, one might wonder if these quantum QED polarization effects could significantly alter the interaction away from the assumed Coulombic potential. At close separation distances $r$ between (point) charges $q$ and $q^{\prime}$, for $r < < \hbar /m_{e} c \approx 10^{ - 12} m$, the interaction potential energy (including virtual $e^{ - } e^{ + }$ screening) is given by ^24,25^

20 $$U(r) = \frac{{qq^{\prime}}}{{4\pi \varepsilon_{o} r}}\left\{ {1 + \frac{2\alpha }{{3\pi }}\left[ {\ln \left( {\frac{\hbar }{{m_{e} cr}}} \right) - \frac{5}{6} - \ln \gamma } \right] + {\rm O}(\alpha^{2} ) + ...} \right\}$$

where the fine structure constant $\alpha \approx 1/137$ and $\gamma \approx 1.781$. From Eq. (20) one finds that

21 $$U(2R_{e} ) = \frac{{qq^{\prime}}}{{8\pi \varepsilon_{o} R_{e} }}\left\{ {1 + 0.024} \right\}$$

where the factor of $0.024$ arises from these virtual $e^{ - } e^{ + }$ screening effects. Thus, inclusion of virtual $e^{ - } e^{ + }$ fluctuations would increase the value in Eq. (19) by ~ 2%. In this publication we shall ignore all virtual $e^{ - } e^{ + }$ screening effects.

From Eqs. (16) and (19) one finds that

22 $$\tau_{CI} = 9.2 \times 10^{ - 13} s$$

which, if Eq. (17) holds, gives the glass transition time

23 $$t_{G} = 3.2 \times 10^{ - 11} s.$$

In the glassy phase it is necessary that the time scale for $e^{ - } e^{ + }$ pair production be much, much smaller than $t_{G}$. The Borsellino formula for the creation of $e^{ - } e^{ + }$ pairs via triplet production^19^ at $T_{G}$ (corresponding to a reduced initial photon energy of $k = k_{B} T_{G} /m_{e} c^{2} = 1.8 \times 10^{7}$) has a total cross-section of $\sigma = 2.7 \times 10^{ - 30} m^{2}$, therefore, the characteristic time for triplet production is

24 $$t_{e + e - } = \frac{1}{\sigma nc} = 5.2 \times 10^{ - 38} s$$

using a number density of $n = 2.4 \times 10^{58} /m^{3}$. As required $t_{e + e - } < < t_{G}$, namely, an $e^{ - } e^{ + }$ pair is created in the glassy phase as soon as sufficient space becomes available during this accelerated expansion of the Universe.

### *Transition between the glassy phase and*$\Lambda CDM$

As $T$ decreases below $T_{G}$ the spacing between adjacent charges increases and, therefore, the potential energy density decreases as

25 $$\psi (T) = \psi_{G} \left( {\frac{{2R_{e} }}{l}} \right)^{4} = \psi_{G} \left( {\frac{T}{{T_{G} }}} \right)^{4} ,\;T \le T_{G}$$

where $l$ is the average spacing between charges. The form taken in Eq. (25) arises because $\psi_{G} \sim (2R_{e} )^{ - 4}$ in Eq. (19). From Eq. (11), at a given temperature, $l$ is determined from

26 $$l = \frac{\hbar c}{{k_{B} T(0.183)^{1/3} }}.$$

It is readily shown, using Eqs. (14) and (26), that the second equality in Eq. (25) follows.

At $T \le T_{G}$ the kinetic energy density $K$ that contributes to the total energy density of intergalactic space is also required. For a particle of radius $R_{e}$ the time for this particle to diffuse its own radius, due to Brownian motion, is given by^26^

27 $$\tau_{D} = \frac{{R_{e}^{2} }}{2D} = \frac{{3\pi \eta R_{e}^{3} }}{{k_{B} T}}$$

where $D$ is the diffusion coefficient and $\eta$ is the solvent viscosity. For our system the average velocity is therefore given by

28 $$\overline{v} = \frac{{R_{e} }}{{\tau_{D} }} = \frac{{k_{B} T}}{{3\pi \eta R_{e}^{2} }}.$$

Near a glass transition the viscosity is divergent according to^26,27^

29 $$\eta = \eta_{0} \exp \left[ {\frac{1.15\phi }{{\phi_{m} - \phi }}} \right]$$

where $\phi$ is the volume fraction at a given temperature $T < T_{G}$ while $\phi_{m}$ is the packing fraction for the glassy phase ($\phi_{m} = 0.52$ for a simple cubic structure). As the spacing $l$ between particles increases then the volume fraction changes according to

30 $$\phi = \frac{{V_{sphere} }}{{l^{3} }} = \frac{4}{3}\pi \left( {\frac{{R_{e} }}{l}} \right)^{3}.$$

Note: as $\phi \to \phi_{m}$ then $\eta \to \infty$ and $\overline{v} \to 0$, hence, $K \to 0$ as required. In Eq. (29) $\eta_{0}$ is the viscosity far from the glass transition, namely, the viscosity of a very dilute gas. For a hard sphere non-interacting gas^28^ (p. 545)

31 $$\eta_{0} = \frac{{0.553\sqrt {m_{e} k_{B} T} }}{{4\pi R_{e}^{2} }}.$$

Finally, the kinetic energy density can be calculated from

32 $$K = \frac{{m_{e} \overline{v}^{2} }}{{2l^{3} }}$$

where $\overline{v}$ is determined from Eqs. (28)–(31) and $l$ from Eq. (26). The calculation of $K$, in Eq. (32), is an approximation that assumes that the “hard sphere” nature of the electron and positron and, therefore, the divergent viscosity $\eta$ (Eq. (29)), predominantly determines the behavior of $K$. Improvements to this model would need to take into account the interactions between electrons and positrons; such improvements would undoubtedly give rise to a far more complicated form for $K$.

According to Eq. (4) the acceleration-deceleration transition corresponds to $w = P/\Pi = - 1/3$ or, equivalently,

33 $$\beta K = \psi ,\;{\text{with}}\,\beta = 2$$

using Eqs. (5)–(6). If one solves Eqs. (25)–(33) for the transition temperature then one finds that

34 $$T = 2T_{G} \left[ {\frac{3}{4\pi }\frac{{\phi_{m} }}{{1 - \frac{2.3}{{\ln (A/\beta )}}}}} \right]^{1/3}$$

where the constant

35 $$A = 18\;(0.553)^{2} (0.183)^{1/3} \frac{{R_{e}^{4} \psi_{G} }}{\hbar c} = 0.0025.$$

Hence, the acceleration-deceleration transition temperature

36 $$T_{T} = 0.96 \times 10^{17} K$$

where the potential energy density

37 $$\psi_{T} = \psi (T_{T} ) = 1.3 \times 10^{50} J/m^{3}.$$

Cosmic inflation is expected to cross-over to the $\Lambda CDM$ model when $w = 1/3$ corresponding to the equation of state for photons and relativistic fermions^20^. In the scalar field description this occurs when

38 $$K = 2\psi$$

i.e. $\beta = 0.5$ and, therefore, from Eq. (34) the cross-over temperature

39 $$T_{X} = 0.94 \times 10^{17} K$$

and cross-over potential energy density

40 $$\psi_{X} = \psi (T_{X} ) = 1.2 \times 10^{50} J/m^{3}.$$

It can readily be shown, from Eqs. (26)–(32), that the kinetic energy density is given by

41 $$K(T) = \frac{{k_{B} T}}{{9(0.553)^{2} R_{e}^{3} }}\left( {\frac{T}{{T_{G} }}} \right)^{3} B(T)$$

where the function

42 $$B(T) = \exp \left[ { - \frac{2.3}{{(T_{G} /T)^{3} - 1}}} \right].$$

Figure 2 provides a plot of $\psi (T)$ and $K(T)$ versus $T$, determined from these calculations, using Igor Pro 4.09. In this calculation we ran into numerical issues, that arise when $(T_{G} /T)^{3} - 1 \approx 0$, in the calculation of $B(T)$ for $T$ very close to $T_{G}$. These numerical issues were avoided by assuming that $B(T) = 0$ when $T > 0.96T_{G}$ while, at lower temperatures ($T \le 0.96T_{G}$), $B(T)$ is described by Eq. (42).

## Discussion

A physical mechanism that gives rise to cosmic inflation has never previously been identified. The scalar field $\varphi$ is normally an unknown and the form for the potential energy density $\psi (\varphi )$ can only be surmised. Invariably, for a particular model of $\psi (\varphi )$, this function contains a number of adjustable parameters that are fine tuned in order that the model gives rise to cosmic inflation for a sufficient number of e-folds (Eq. (8)). Additionally, questions arise as to how inflation ends, as well as, how the energy contained in the inflaton $\varphi$ decays and is converted to particles in the Standard Model, at thermodynamic equilibrium, such that the $\Lambda CDM$ model can proceed as normal, giving rise to BBN and the CMB, etc. Planck collaboration 2013 results have ruled out many forms for $\psi (\varphi )$ as they do not conform with a plateau potential^17^. These issues have led to an intense debate as to whether or not cosmic inflation, in the form proposed in the literature, can account for the isotropy, homogeneity, as well as, magnitude and distribution of thermal fluctuations $\delta T/T\sim 10^{ - 5} - 10^{ - 4}$ within the CMB^16,29^. In comparison to these earlier cosmic inflation models, the eBse model discussed here, does not suffer from any of these drawbacks. There are no adjustable parameters in the eBse model. The inflaton has been identified to be temperature $T$, in this model, where the inflaton potential energy density $\psi (T)$ (Fig. 2) is a plateau potential and can be explicitly calculated. Cosmic inflation, with exponential acceleration, occurs naturally above the glass transition temperature $T_{G}$ where the eBse model “Gracefully exits” to the $\Lambda CDM$ model below a temperature $T_{X}$.

In summary, in earlier work^8,9^ we have shown how the eBse model quantitatively explains many features attributed to Dark Energy at small redshifts, of order $z \approx 0 - 2$, and *low intergalactic densities*, with baryon number density $n \approx 1/(4\,m^{3} )$. If the eBse model is to provide a valid description of the Universe then, at early times, a crucial test will be the behavior that this model exhibits at *very high plasma densities* ($n \approx 10^{58} \,m^{ - 3}$) where the separation distance between electrons and positrons is of order $2R_{e}$. In the current publication we demonstrate that in this high density region the eBse model undergoes exponential acceleration due to a constant potential energy density $\psi (T)$ (Fig. 2), akin to CI, caused by the non-equilibrium conversion of photons to $e^{ - } e^{ + }$ pairs above a glass transition temperature of $T_{G} = 1.06 \times 10^{17} K$ (14). This model naturally crosses over to the $\Lambda CDM$ model below a temperature $T_{X} = 0.94 \times 10^{17} K$ (39). $\psi (T)$ is a plateau potential in conformity with Planck collaboration 2013 analysis of the CMB anisotropy^16,17,30^. There are no adjustable parameters in the eBse model, however, this model for CI is still incomplete as photonic transport in the glassy phase is not yet understood, the presence of other Standard Model particles has not been considered, and quantum fluctuations, that may account for thermal fluctuations $\delta T/T$ in the CMB, remain to be studied.

An anonymous reviewer has pointed out that the assumption of a point-like electron in QED is a historical misunderstanding, as there are no point-like states in Quantum Field Theory, and the notion of the size of a quantum object, in general, can only be provided by its cross-section in specific processes. It is therefore an open question whether or not the electron cross section, at the energy scales of relevance for the onset of inflation (i.e. before inflation has started), is sufficiently large to trigger the proposed mechanism where additionally the effective electron radius remains large enough for the required number of e-foldings during the inflationary period.

In this manuscript we have chosen to study an over-simplified model where the “Universe” consists of photons, electrons, and positrons at very high densities. This over-simplified model allows one to identify a generic mechanism that naturally gives rise to cosmic inflation while allowing the explicit calculation of $T_{G}$, $\psi_{G}$, $\psi (T)$, and $K(T)$. In this generic mechanism, that gives rise to cosmic inflation, all that is necessary is that the particle under consideration possess a finite, non-zero radius. Thus, if quarks possess a finite, non-zero radius, cosmic inflation will occur during the quark/anti-quark creation process (from photons) above the corresponding glass transition temperature. If the quark radius is similar to the electron radius, assumed in Eq. (1), then the quark/anti-quark $\psi_{G}$ is likely to dominate the electron/positron $\psi_{G}$ by perhaps a factor of ~ 100 because the strong nuclear force is a factor of ~ 100 larger than the electromagnetic force.

### Supplementary Information

Supplementary Information.

## Data Availability

All data generated or analyzed during this study are included in this published article.
