# Supplementary material for: Proposed physical mechanism that gives rise to cosmic inflation
Source: Sci Rep. 2023 Dec 9;13:21798. doi: 10.1038/s41598-023-49106-0 (PMC10709372; doi:10.1038/s41598-023-49106-0)
Supplement: Supplementary file 1 — Supplementary Information. [file 41598_2023_49106_MOESM1_ESM.pdf]

Proposed physical mechanism that gives rise to cosmic inflation

Bruce M. Law*
Department of Physics
116 Cardwell Hall
Kansas State University
Manhattan, KS 66506-2601
*bmlaw@phys.ksu.edu

Supplementary Material: Interrelationship of eBse model and QED

The ΛCDM model indicates that the Standard Model for particle physics only describes ~5% of the Universe (the baryons), hence, any model that is capable of describing DE and/or CDM must necessarily be an extension of the Standard Model. A requirement for any extension to the Standard Model is that this new model not conflict with any experimental data that is generally accepted by the scientific community and which is successfully described by the Standard Model. This requirement imposes severe restrictions on the types of extensions to the Standard Model that are possible. The eBse model is an extension of QED, the interaction between photons and electrons. QED forms part of the Standard Model. In QED the electron is assumed to be a point particle possessing zero radius ($R_e \to 0$) [1]. This point particle assumption, for the electron, leads to various divergences that must be renormalized away, whereby infinite quantities are replaced by finite measurable quantities. In particular, when a (stationary) electron and positron annihilate to give two photons, to conserve energy and momentum, then [2]

$$2(m_b c^2 + Qse) \to 2m_e c^2 , \qquad \text{(S1)}$$

where m_b is the bare or Dirac mass that arises in the Dirac equation and Qse is a divergent quantum mechanical self-energy that diverges logarithmically with R_e [1] and arises from virtual photons and virtual particles that flit in and out of existence while interacting with the electron or positron. The arrow, in equation (S1), implies that the divergent quantities on the left hand side are replaced by the finite quantity on the right hand side.

A divergent quantity that does not appear explicitly in equation (S1) is the Born self-energy, corresponding to the energy contained in the electric field that surrounds the electron (or positron). This Born self-energy has the specific form [3,4]

$$U_e^{Born} = \frac{q^2}{8\pi\varepsilon_o R_e} , \qquad \text{(S2)}$$

where q is the charge and ε_o is the vacuum permittivity. In QED, to eliminate this divergent quantity, it is assumed that U_e^{Born} is subsumed or contained within m_e [5]. In the 1950s when QED was being developed there was little choice but to make these renormalization assumptions, in order to eliminate diverging quantities, because measurements of the interactions of electrons, positrons, and photons were rather rudimentary at that time. Both Dirac [6] and Feynman [7] expressed reservations about this renormalization process. QED is currently the most accurate theory in physics where, for example, theory and experiment for the electron magnetic moment μ / μ_B agree to eleven decimal places [8]. The ***difference*** between theory and experiment for the electron magnetic moment (in the 12th decimal place) gives [8]

$$\left|\Delta(\mu / \mu_B)\right| = \left|(\mu / \mu_B)_{theory} - (\mu / \mu_B)_{\exp}\right| \approx 1_{0.28}^{0.77} \times 10^{-12} , \qquad \text{(S3)}$$

where 0.77 (0.28) represents the error in the theoretical calculation (experimental measurement). Equation (S3) can be used to estimate an ***upper bound*** for the electron radius [9]

$$R_\mu^{Upper} \approx 1\times10^{-18}\,m\,, \tag{S4}$$

where the actual electron radius $R_e < R_\mu^{Upper}$.

The eminent success of QED has meant that few scientists today question the underlying foundations of QED. However, in Physics, there are a number of foundational principles that all physicists agree upon and any discrepancies from these foundational principles potentially point to new physics provided that these discrepancies can be resolved. In particular, Physics ***must*** be self-consistent across all subfields, therefore, findings in one subfield may influence the understanding of a different subfield. Additionally, conservation laws, such as the conservation of energy, are expected to hold across all subfields of Physics.

There are two foundational flaws in QED that are not widely recognized:

(i) QED is inconsistent with the treatment of ions in soft matter physics. In soft matter physics the solubility of ions in solution is governed by the Born self-energy (equation (S2)) [4]. Therefore, to ensure that QED and soft matter physics are self-consistent with each other the Born self-energy, U_e^{Born}, cannot be subsumed within m_e for an electron. Namely, m_e and U_e^{Born} must be treated as two separate, independent entities.

(ii) In QED it is assumed that an electron has energy $m_e c^2$ at the point of annihilation with a positron. Additionally, this electron also possesses the ***same*** energy as a free particle (at infinite separations). This cannot be correct because, under such circumstances, energy is not conserved. A free electron ***must*** possess a greater energy than an electron that is about to be annihilated because work must be done against the Coulomb force in order to separate an electron and a positron from a separation distance of R_e out to infinity.

These two foundational flaws in QED can only be resolved if the electron possesses a finite, non-zero radius R_e where the Born self-energy U_e^{Born} is treated as a separate and distinct quantity from m_e. Specifically, if one considers an electron and positron separated by distance r then upon incorporating the Coulomb interaction as well as the Born self-energy, the total energy of interaction between these two particles is [10]

$$E^{tot}(r) = 2\left(m_b c^2 + Qse + \frac{q^2}{8\pi\varepsilon_o R_e} \right) - \frac{q^2}{4\pi\varepsilon_o r}\,. \tag{S5}$$

Upon electron-positron annihilation ($r \to R_e$), the Born self-energy term and the Coulomb interaction cancel in equation (S5), and consequently

$$E^{tot}(r \to R_e) = 2(m_b c^2 + Qse) = 2m_e c^2\,, \tag{S6}$$

which is a justification for the renormalization process in equation (S1).

In the other limit, for free particles,

$$E^{tot}(\mathrm{r}\to\infty)=2\left(m_e c^2+\frac{q^2}{8\pi\varepsilon_o R_e}\right), \tag{S7}$$

and $m_e c^2$ and U_e^{Born} are separate independent terms, as occurs for other ions, where U_e^{Born} can alternatively be viewed as arising from the work done against the Coulomb force in separating the electron and positron from R_e to infinity. Thus, the (non-local) energy is conserved in the eBse model. In earlier publications [10,11] we have shown how U_e^{Born} quantitatively describes DE, within error bars, with no free parameters.

The final issue that requires addressing is "Will the finite electron radius, used in the eBse model, perturb the agreement between theory and experiment in QED? [12,13]". The upper bound for the electron radius, R_μ^{Upper} (equation (S4)), implies that for any ***smaller*** electron radius, theory and experiment for μ/μ_B will continue to agree, where the effects of this smaller electron radius will be hidden within the error bars of $|\Delta(\mu/\mu_B)|$ (equation (S3)). In the eBse model the electron radius is assumed to be $R_e=1.9\times10^{-20}m$ (1), whose value was derived from electron-positron collisions [11,14]. For this value of $R_e(<R_\mu^{Upper})$ one finds that $|\Delta(\mu/\mu_B)|\approx10^{-15}$ (i.e. theory and experiment for μ/μ_B would only disagree at the 15th decimal place for this particular value of R_e) and the effects of this finite-sized electron radius are hidden within the error bars contained within equation (S3).

In summary, the eBse model ensures that (a) the non-local energy is conserved for the electron, (b) the solubility of ions and electrons in solution can be explained using the same physical phenomenon, namely, the Born self-energy (equation (S2)), and (c) the value assumed for the electron radius (equation (1)) does not conflict with experimental measurements and QED theory for the electron magnetic moment.

References

1 Weisskopf, V. F. The development of field theory in the last 50 years. *Physics Today* **34**, 69-85 (1981).

2 Bjorken, J. D. & Drell, S. D. *Relativistic quantum mechanics*. (McGraw-Hill, 1964).

3 Feynman, R. P., Leighton, R. & Sands, M. *The Feynman Lectures on Physics*. Vol. II Chaps. 8 & 28 (Addison-Wesley, 1964).

4 Israelachvili, J. N. *Intermolecular and surface forces*. 3rd edn, (Academic Press, 2011).

5 Heitler, W. *The quantum theory of radiation*. 3rd edn, (Dover, 1984).

6 Dirac, P. A. M. The evolution of the physicist's picture of nature. *Scientific American* **208**, 45 (1963).

7 Feynman, R. P. *QED: The strange theory of light and matter*. (Princeton University Press, 1985).

8 Gabrielse, G. The standard model's greatest triumph. *Physics Today* **December**, 64-65 (2013).

9 Gabrielse, G., Hanneke, D., Kinoshita, T., Nio, M. & Odom, B. New determination of the fine structure constant from the electron g value and QED. *Phys. Rev. Lett.* **97**, 030802 (2006).

10 Law, B. M. Electron Born self-energy model for Dark Energy. *Phys. Sci. Forum* **2**, 9 (2021).

11 Law, B. M. Cosmological consequences of a classical finite-sized electron model. *Astrophys Space Sci* **365**, 64 (2020).

12 Thumm, U. Private communication (2020).

13 Peebles, P. J. E. Private communication (2020).

14 Bourilkov, D. Hint for axial-vector contact interactions in the data on $e^+e^- \rightarrow e^+e^-(\gamma)$ at center-of-mass energies 192-208 GeV. *Phys. Rev. D* **64**, 071701R (2001).
